# Intestinal Malrotation With Colon Cancer: A Rare Cause of Obstruction in Adults

**DOI:** 10.7759/cureus.65688

**Published:** 2024-07-29

**Authors:** Ammar Aleter, Reham A Taha, Mahwish Khawar, M Yousif, Mohamed A Kurer

**Affiliations:** 1 Colorectal Surgery Unit, Department of Surgery, Hamad Medical Corporation, Doha, QAT; 2 College of Medicine, Qatar University, Doha, QAT

**Keywords:** midgut malrotation, right colon cancer, large intestinal obstruction, adult intestinal malrotation, colorectal cancer surgery

## Abstract

Intestinal malrotation is an infrequent congenital anomaly. Its presentation in adults is rare, and it is usually discovered incidentally. This article presents an extremely rare case of an adult patient presenting with obstructing colon cancer associated with intestinal malrotation. This is the ninth case to be published in the past 40 years. After proper resuscitation and imaging, an open resection was performed for the patient due to unresolving obstruction and significant abdominal distention. This case highlights the rarity of colon cancer in a malrotated gut and the importance of preoperative evaluation of the unique anatomy before surgical intervention. It also discusses the possible surgical options for such patients with obstruction due to colon cancer causing suboptimal circumstances for both minimally invasive intervention and reestablishment of bowel continuity.

## Introduction

Intestinal malrotation is a congenital anomaly presenting commonly in the first month of life with acute bowel obstruction and midgut volvulus [1]. It is unusual to be found in adults and is mostly discovered incidentally during surgery when explored for other etiologies [2]. Some patients might present with anemia, weight loss, or constipation; however, an emergency presentation with obstruction or perforation is extremely rare [3]. Diagnosing such patients and managing them needs experience and careful planning to avoid any iatrogenic injury, especially with the different types of malrotation and possible vascular anomalies [4]. This report describes a case of malrotation with ascending colon cancer causing large bowel obstruction that warranted an emergency operation. The unusual presentation was discussed, along with all investigations and images needed to provide the best possible management for the patient.

## Case presentation

A 50-year-old Nepalese male presented to the emergency department complaining of colicky abdominal pain of three weeks duration. Four days prior to presentation, the pain increased in severity and was associated with abdominal destination, vomiting, and obstipation. The patient's vitals were stable, and his examination was remarkable for a distended abdomen which was non-tender. A digital rectal exam revealed an empty rectum with no palpable masses. Laboratory investigations revealed anemia and no electrolyte imbalance, and his inflammatory markers were within normal limits. His tumor markers CA 19-9 (carbohydrate antigen 19-9) and CEA (carcinoembryonic antigen) were significantly raised (Table 1). The patient did not undergo any type of screening prior to his presentation.

**Table 1:** Patient's laboratory investigations after admission with intestinal obstruction. CA 19-9: carbohydrate antigen 19-9; CEA: carcinoembryonic antigen

Laboratory parameters	Patient values	Reference range
White blood cells	12.7×10^3^/uL	4-10×10^3^/uL
Hemoglobin	8.2 gm/dL	13-17 gm/dL
Creatinine	134 umol/L	60-106 umol/L
Albumin	27 gm/L	35-50 gm/L
CA 19-9	1,756.0 U/mL	0.0-27 U/mL
CEA	477.0 ug/L	0-2.5 ug/L

**Figure 1:**
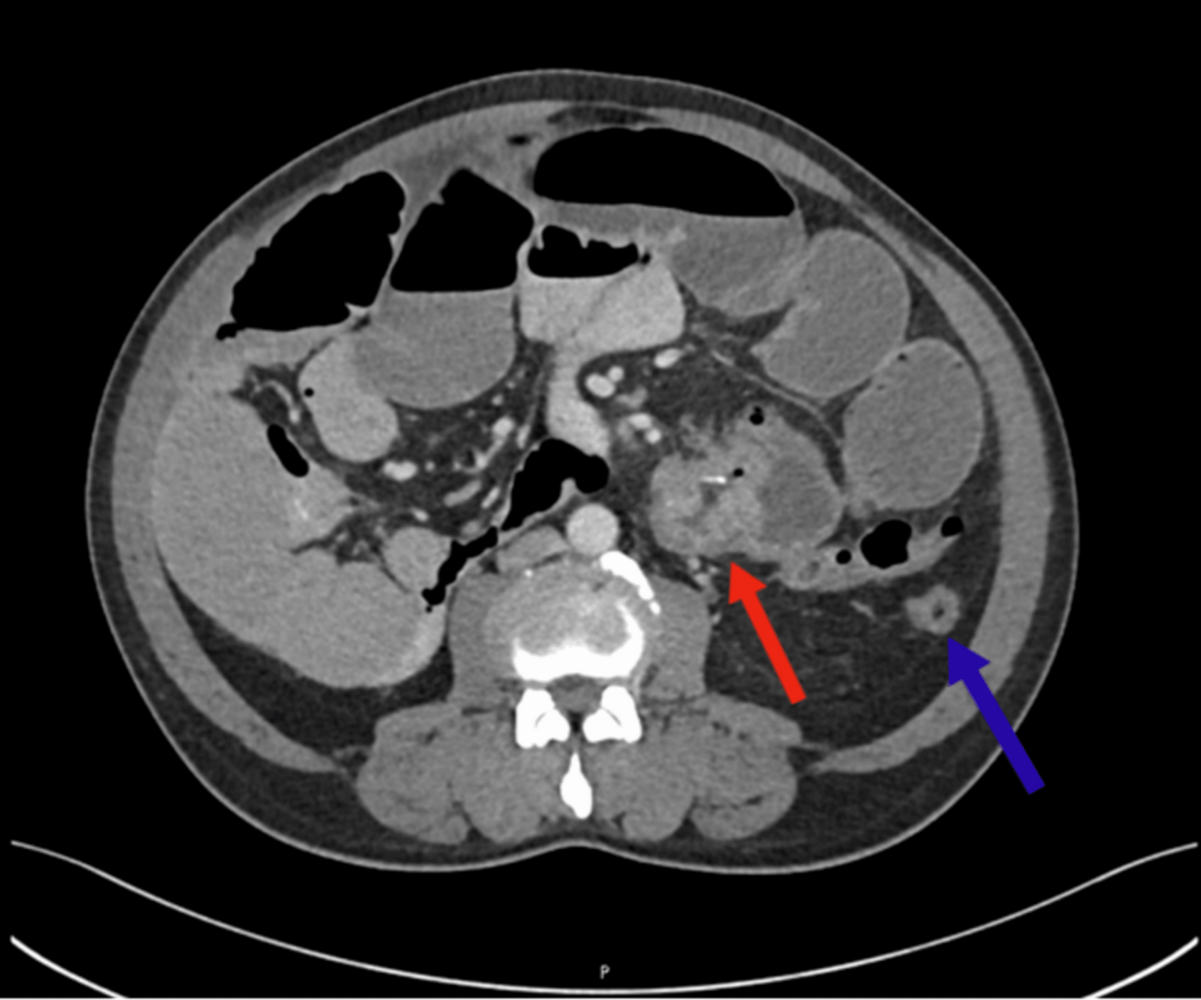
Axial view on CT of the abdomen showing both the obstructing ascending colon tumor (red arrow) and descending colon (blue arrow) on the left side of the abdomen. CT: computed tomography

**Figure 2:**
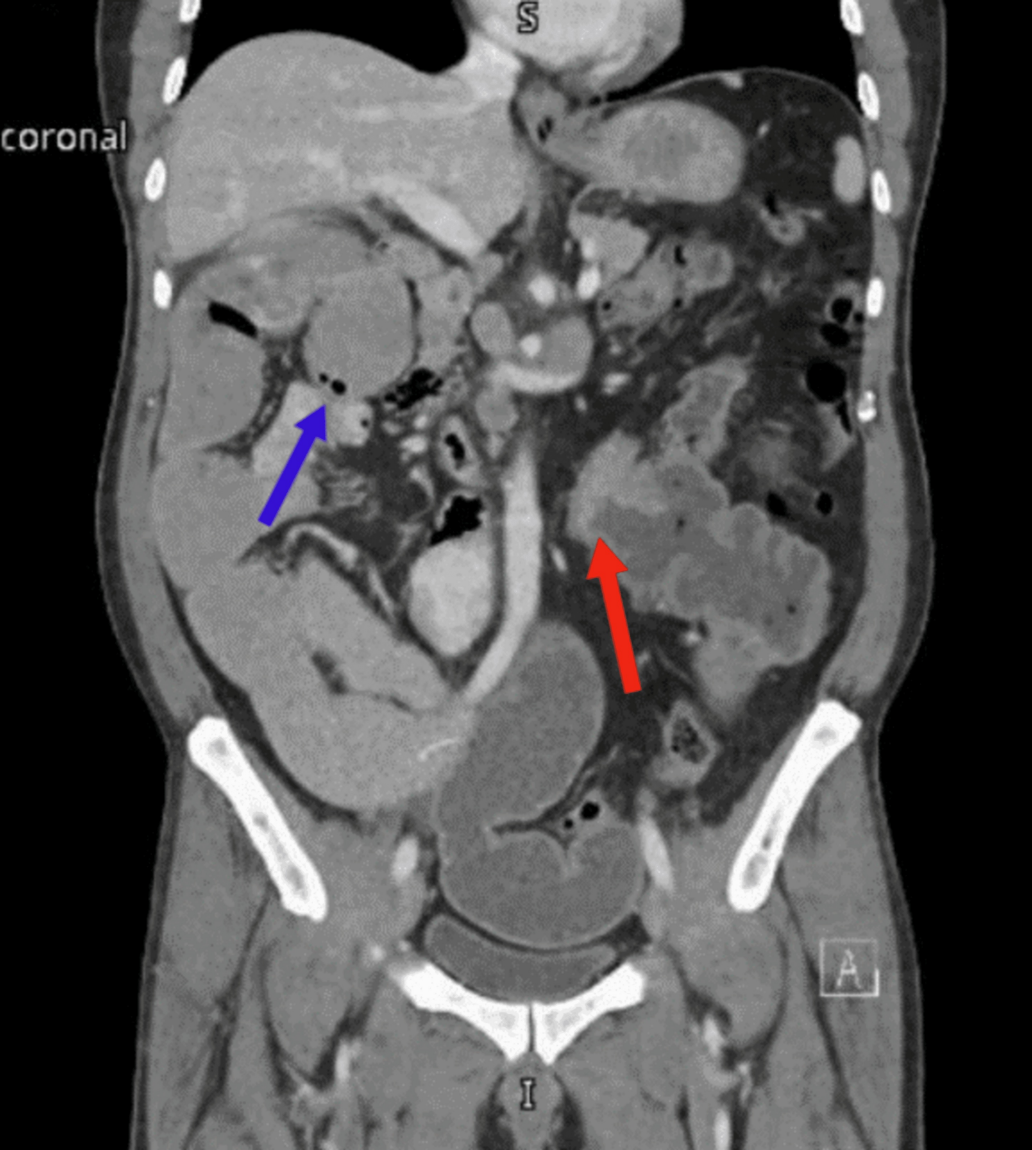
Coronal view on CT of the abdomen showing the obstructing ascending colon tumor (red arrow) on the left side of the abdomen and the distended small bowel loops on the right side of the abdomen including the duodenojejunal junction (blue arrow). CT: computed tomography

An abdominal computed tomography (CT) scan was done that showed a malrotated bowel with the cecum seen in the left upper quadrant associated with dilated small bowel loops. On top of that, a circumferential ascending colon mass was seen causing intestinal obstruction with multiple adjacent enlarged lymph nodes extending towards the base of the mesentery. A 5×5 cm solitary left liver lobe metastasis was also noted (Figure 1 and Figure 2). 

The patient was admitted, resuscitated, and placed on NGT to decompress his bowel loops. Over a course of 24-48 hours, the clinical picture of the patient remained unchanged, as the ascending colon mass was quite constricting, causing significant obstruction. Therefore, the decision to perform an emergency laparotomy was made.

**Figure 3:**
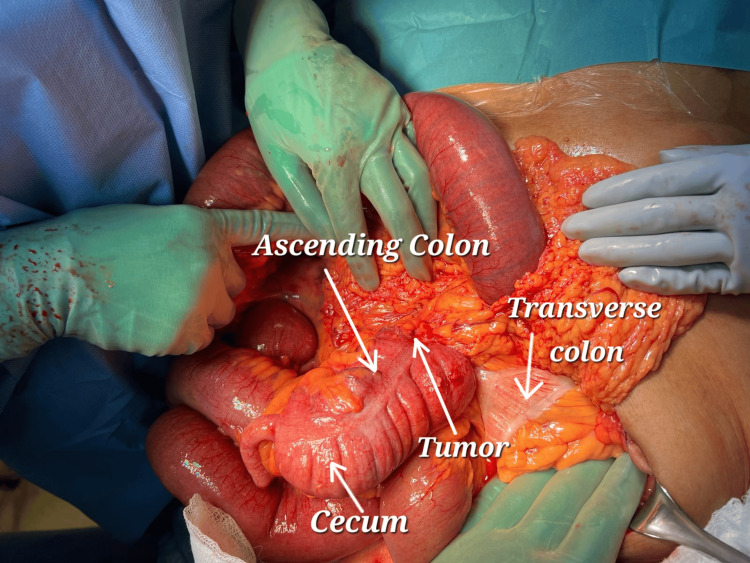
Intraoperative findings during exploration. Notice the nonrotational type of malrotation with the obstructing ascending colon tumor in the middle of the abdomen, next to the transverse and descending colon on the left side of the abdomen.

**Figure 4:**
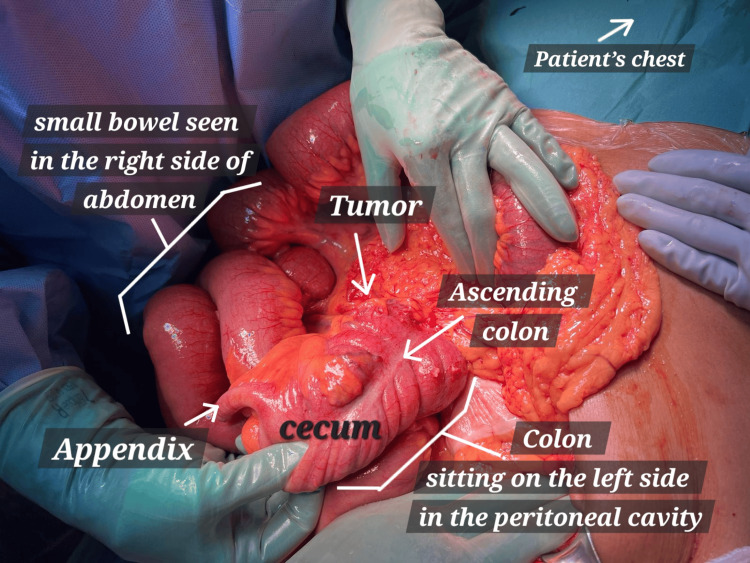
Another view during the exploratory laparotomy showing the obstructing tumor on the left side of the abdomen and the distended small bowel loops (malrotated) on the right side.

Upon exploration, a nonrotational type of malrotation was noticed, with the small bowel on the right and the colon on the left side of the abdomen. The duodenal C-loop was absent and the duodenojejunal junction was on the right side of the abdomen. There were multiple bands from the lateral abdominal wall towards the appendix, cecum, and ascending colon, fixing them in the middle of the abdomen. The ascending colon mass was causing significant obstruction and dilatation of the cecum and small bowel (Figure 3 and Figure 4). 

Adhesiolysis was performed, and the cecum and ascending colon were freed, followed by a right hemicolectomy. The patient also required vasopressor support intraoperatively as a result of a prolonged period of dehydration, acidosis, and obstruction. In addition, the need for extensive adhesiolysis together with resection of the large tumor extended the duration of the procedure. In light of the aforementioned scenario, the decision was made to bring a double barrel stoma instead of primary anastomosis.

The patient was transferred to the ICU postoperatively, where he was kept for a couple of days before being shifted to the ward. The patient had a slow but smooth recovery postoperatively and was discharged home in stable condition 10 days after surgery. The final surgical pathology report showed a 5×3×2 cm tumor located in the ascending colon, invading the visceral peritoneum. Histopathology revealed a moderately differentiated invasive adenocarcinoma (pT4a, pN2b), and nine out of 17 lymph nodes examined were positive. A staging CT showed a solitary left lobe liver metastasis. The patient's case was discussed in the colorectal multidisciplinary meeting, where a decision of adjuvant chemotherapy followed by liver resection of the solitary liver metastasis was made.

## Discussion

Midgut malrotation is caused by a congenital incomplete or nonrotational movement of the primitive intestinal loop around the axis of the superior mesenteric artery (SMA) during intrauterine life. Malrotation anomalies can happen in the form of nonrotation, incomplete rotation, reversed rotation, and paraduodenal hernias depending on the embryonic phase of development in which the arrest of rotation occurs [1]. The nonrotation subtype occurs due to the failure of the midgut to rotate, causing the small bowel to lie on the right hemiabdomen with the colon laying on the left [4].

In general, intestinal malrotation occurs in approximately one in 6,000 live births. Nevertheless, adult presentation is rare since up to 80% of malrotation cases are discovered in infants within the first month of life [5]. The majority of quiescent malrotations in adults are detected incidentally on a CT scan. It shows both intestinal malpositioning and the associated congenital and vascular anomalies. Since it is mostly found incidentally in adults, the prevalence of each subtype of malrotation is not known. The present case seemed to resemble the nonrotation type, which is the most frequent one [1,3].

The occurrence of colon cancer with intestinal rotation is rare. From 1970 to 2024, the literature documented 62 cases of intestinal malrotation with colon cancer, out of which 51 were from Japan [6,7]. Most of the cases were on the right side of the colon (64%) [8]; however, patients presenting with intestinal obstruction due to the tumor and the need for emergency surgery were documented in only eight cases [2,3]. Table 2 shows the reported cases of malrotation presenting with obstruction due to colon cancer. Laparoscopic resection was done in 19 cases (30.6%) [6,7]. To our knowledge, this is the first case report documented in the Middle East.

**Table 2:** Adult patients with malrotation and colon cancer causing intestinal obstruction. CT: computed tomography

Author	Country	Sex	Age	Type of intestinal malrotation	Location of malignancy	Diagnosis method	Intervention
Nakayama et al., 2016 [2]	Japan	M	63	IA (nonrotation)	Descending colon	CT scan	Resection and anastomosis
Gilbert et al., 1990 [9]	United Kingdom	M	55	IIIA (normal duodenal rotation, no colonic rotation)	Splenic flexure	Intraoperative	Defunctioning transverse colostomy
Yokota et al., 1995 [10]	Japan	M	66	IA (nonrotation)	Rectum	Intraoperative	Hartmann's procedure
Shimanuki et al., 1988 [11]	Japan	M	73	IA (nonrotation)	Cecum	Barium X-ray	Resection and anastomosis
Mitani et al., 2006 [12]	Japan	F	76	IIID (paraduodenal hernia)	Transverse colon	CT scan	Resection and anastomosis
Fukuhara et al., 2010 [12]	Japan	F	76	IA (nonrotation)	Cecum	Intraoperative	Resection and anastomosis
Itatani et al., 2009 [13]	Japan	M	61	Malrotation	Transverse colon	CT scan	Resection and anastomosis
Kim et al., 2011 [14]	Korea	M	63	IA (nonrotation)	Ascending colon	CT scan	Resection and anastomosis
Current case	Qatar	M	50	IA (nonrotation)	Ascending colon	CT scan	Resection and diverting stoma

If surgical intervention is indicated for such patients with rare disorders, multiple modifications are needed to improve the outcome. One of these modifications is to confirm the presence of any arterial or venous variations on enhanced CT before embarking on surgery. This would help avoid any vascular injury, mainly during dissection over the SMA and SMV [2]. Also, freeing Ladd's band and any other congenital bands holding the colon is essential for mobilization and resection [4]. This step was challenging in our case, especially with colonic obstruction and extensive dilatation. After proper oncological resection and lymphadenectomy, the next step is to decide whether to join the bowel or to create a stoma for the patient.

Joining the bowel was applicable in all eight cases of malrotation with obstructing colon cancer, except for two cases. Gilbert et al. opted for a diverting loop colostomy to deflate the bowel, and resection of the splenic flexure tumor was completed after appropriately staging the patient in an elective condition [9]. On the other hand, Yokota et al. discovered the malrotation intraoperatively during exploration for an upper rectal cancer causing intestinal obstruction; Hartmann's procedure was done [10]. In our case, the patient was dehydrated and obstructed for four days. The additional need for adhesiolysis in order to free the congenital bands and thus achieve proper oncological resection prolonged the procedure. Not only did the surgery time become longer, but the requirement of intraoperative vasopressor support directed our decision towards creating a diverting stoma instead of an anastomosis to avoid any possibility of leakage.

## Conclusions

Malrotation of the intestine, though commonly a pediatric problem, can infrequently present in adults. While most of these cases in adults are discovered incidentally, some might present with anemia, constipation, abdominal pain, and rarely intestinal obstruction. To our knowledge, this rare case is the first to be reported in the Middle East region.

As for the management, laparoscopic intervention may be performed in many of these patients; however, for our patient with intestinal obstruction and gut malrotation, we preferred the open approach for better delineation of the intestinal aberrant anatomy as well as the lack of intra-abdominal space occupied by the extensively distended bowel. We felt that both the above factors would have precluded a safe resection of a malignant disease.
